# Planning for the future: cancer incidence projections in Switzerland up to 2019

**DOI:** 10.1186/1471-2458-14-102

**Published:** 2014-02-01

**Authors:** Elisabetta Rapiti, Sandrine Guarnori, Bert Pastoors, Raymond Miralbell, Massimo Usel

**Affiliations:** 1 Geneva Cancer Registry, University of Geneva, Geneva, Switzerland; 2 Radiation Oncology Department, Geneva University Hospitals, Geneva, Switzerland

**Keywords:** Cancer, Incidence, Population-based cancer registries, Projections

## Abstract

**Background:**

Projections of the national burden of cancer play a key role in planning cancer control programmes and investments. We present projections of cancer incidence rates and cases for the period up to 2015-2019 in Switzerland.

**Methods:**

Projections were based on cancer incidence data estimated from cancer registries for the 1989-2009 periods and demographic projections of the Federal Statistical Office. Age-specific incidence rates were modelled as a function of age, period-birth cohort using NORDPRED.

**Results:**

Up to 2019 the incidence of all cancers combined is expected to decrease slightly for both sexes. Nevertheless, the overall number of cases is predicted to increase. The number of male cancer cases will increase by 30%, from 20005 in 2005-2009 to 25910/year in 2015-2019. For females the number will increase by 20%, from 16913 to 20359/year in 2015-2019. Changes in the population size and structure will be responsible for most of the increase. Among men, the largest increase is observed for melanoma (+54%), thyroid (+45%), non-Hodgkin lymphoma (+43%), and prostate (+37%). Prostate cancer will contribute with 8083 cases, colorectal cancer with 2908 and lung cancer with 2791. For women, cases of lung and oral cavity cancers will increase by +48% and +38%, respectively; those of thyroid by +45% and non-Hodgkin lymphoma by +36%. The sites with the most cancer predicted are breast (5870), colorectal and lung (over 2000 each), melanoma (1341) and corpus uteri (1040). The overall annual cancer burden predicted for 2015-19 is of 46269 new cases in Switzerland.

**Conclusions:**

Substantial investments appear to be needed in Switzerland cancer services to meet and fill absolute increased demand driven by aging population.

## Background

Estimating the rates of cancer at future time-points at a national level is essential to plan for future services and allocation of resources and to help establish and evaluate cancer control programmes.

The main determinants of future burden of cancer in a population are changes in a country’s demography and variations of exposure to cancer risks and preventive factors [1]. As the great majority of cancers occur more commonly in older adults, the aging of the population in developed countries predicts an increase in the absolute number of cancer cases. More difficult is to correctly predict changes in the profile of cancer burden as a result of variations in aetiological factors or proposed interventions. In fact, at present we lack knowledge of the factors that drive trends for most cancers.

Switzerland’s population is older and people live longer than almost anywhere else in the world [2]. At the same time, lifestyle changes today predict a rising of health risks within the Swiss population in the future [3]. Both these factors hold the prospect of an increasing demand on the health system, particularly in dealing with chronic diseases such as cancers. The Swiss health system with its universal coverage, and high levels of access to services, is high-performing with particularly strong hospital care delivery. It will, nevertheless, require adapting to the challenges of the future to ensure an optimal mix between prevention, promotion and treatment.

In this study, we estimated for the first time the incidence for all cancers in Switzerland over the period 2010-2019. We used the age-period-cohort model with a power link function that relies entirely on the extrapolation of the recorded rates in the past and on the forecasted population data.

## Methods

### Incidence Data

For our study we used the cancer incidence figures provided by the National Institute for Cancer Epidemiology and Registration (NICER). NICER regularly estimates national cancer figures based on the data provided annually by the cantonal registries and for those regions of Switzerland not covered by cancer registration the numbers are extrapolated [4].

For our study, we used the number of cases for the years 1985 to 2009 (by 1985 cancer registries covered approximately 50% of the Swiss population). Data were aggregated into five 5-year periods (1985-1989 to 2005-2009) and eighteen 5-year age groups (0-4 to 85+) by sex. We considered all invasive cancers combined and 12 major individual cancer sites according to the Classification of Diseases, 10th Revision/ICD10 codes [5] (Table 1). By subtracting from the number of all cancers combined the number of cases from the 12 specific cancer sites we obtained the category “other cancers”.

**Table 1:** Trend chosen to project the common drift parameter D accounting for the linear component of the trend in period and cohort by cancer site

**Cancer site**	**ICD-10 codes**	**Trend**^**a**^	
		**Male**	**Female**
Oral cavity & pharynx	C00-14	Average	Recent
Stomach	C16	Recent	Average
Colon, rectum	C18-20	Recent	Recent
Lung, bronchus, trachea	C33-34	Recent	Average
Skin melanoma	C43	Average	Average
Breast	C50		3 age classes <50: Average 50-60: Recent 70+: Recent
Corpus uteri, uterus NOS	C54-55		Average
Ovary	C56		Average
Prostate	C61	2005-2009 rate^b^	
Bladder	C67	Average	Recent
Thyroid	C73	Average	Average
Non Hodgkin lymphoma	C82-85, C96	Recent	Recent
Other cancers (All cancers minuscancer sites)	C17, C21-32, C35-42, C45-49, C51-52, C57-60, C62-66,C68-72, C74-81, C86-95, C97	Average	Average

^a^ Average: average trend over entire prediction base; Recent: average trend from last 10 years.

^b^ Constant age specific incidence rates of last observed period (2005-2009).

### Population data

For the demographic evolution of the Swiss population by 2019 we used the reference scenario called "average (A-00-2010)” published in 2010 by the Swiss Federal Statistical Office. This scenario hypothesizes in 2060 a stabilization of the fertility rate at 1.5 children/woman, the life expectancy at 86 years for men and 90 years for women, and a net migration of 23000 people [6].

### Modelling of incidence

To calculate cancer incidence projections we used NORDPRED [7].

As recommended by Moller et al. [8], we used the age-period-cohort model with a power link function. The model is as follows:

$$R_{\mathrm{ap}}={\left(A_{a}+D\cdot p+P_{p}+C_{c}\right)}^{5}$$

where R_ap_ is the incidence rate in age group a and period p, A_a_ is the parameter attributable to the age group a, D is the common drift parameter accounting for the linear component of the trend in period and cohort, P_p_ is the nonlinear effect attributable to the period p, and C_c_ is the nonlinear effect attributable to the cohort c.

We chose the lower age limit as the smallest age group for which all models converged for each cancer site: 35-39 years for men and 20-24 years for women. Projections for age groups below this limit were based on average rates in the last 10 years (the two last periods).

The number of periods in the prediction base was chosen as the greatest, i.e. five 5-years periods.

The parameters of the model for projected rates were derived as described for the Nordic countries [7,8] as follows. The age component A was projected directly. The nonlinear cohort effects C were projected directly for known cohorts and taken equal to the last estimated effect in the model for new cohorts. The non-linear period effects P were taken to equal the last estimated effect in the model for all future periods. The linear drift D was assumed to continue, but with some reduction in the later prediction periods. This allows reducing the impact of current trends in the projections. So, we added D and 0.75D to the first and second new periods.

Moreover, the projection of the drift is influenced by older trends. If there were significant sharp changes in the historical rates, projections based on the entire set of historical rates would be inaccurate. The test for departure from a linear trend consisted of checking the significance of S in the following model:

$$R_{\mathrm{ap}}={\left(A_{a}+D\cdot p+S\cdot p^{2}+C_{c}\right)}^{5}$$

In cases where S was significant, we used only the trend in the most recent 10 years to project the drift component D. Whether average or recent trend was used is shown in Table 1.

In order to take into account recent changes in incidence rates due to modifications in screening behaviours, cancer of the prostate and breast were not based on extrapolation of past incidence trends as such. In many high income countries, including Switzerland, after a dramatic increase in prostate cancer incidence, result of the widespread introduction of prostate-specific antigen (PSA) testing, trends started to stabilize in most recent years [9]. For this reason, we modelled prostate cancer projections assuming that the rates observed in the most recent period, 2005–09, would remain unchanged in future periods [10,11].

To account for the effect of the introduction of organized screening programs for women 50 to 69 in some cantons during the study period, for breast cancer we fitted the period-cohort models separately to three age groups: <50, 50-69 and >69.

We fitted models for nine major cancer sites in males and for 11 in females plus one for “other cancers” for each sex. The projections for “all cancers” were obtained as a sum of the individual site projections. Finally, projected incidence rates were then calculated based on the resulting models. Age-standardized incidence rates were calculated using the European standard population.

The percent change in cancer incidence counts over the projection period was separated into the contribution from change in cancer risk and the contribution from change in demographics (size and age of population). The portion of the change due to change in risk was calculated by subtracting the number of cases that would result from multiplying current incidence by the estimated future population from the estimated number of future cases. Similarly, the portion of the change due to change in population was calculated by subtracting the current number of cases from the number of cases that would result by multiplying the current incidence and the estimated future population.

## Results

**Figure 1:**
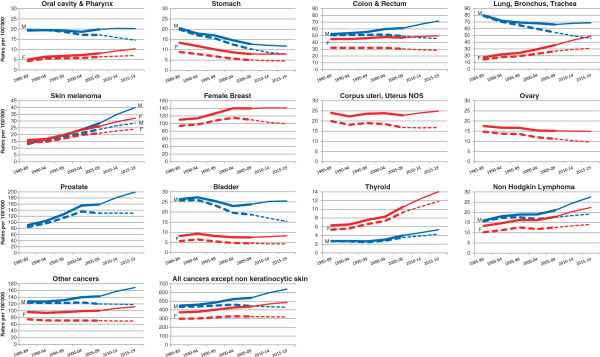
**Actual and projected rates for all cancer and individual cancer sites.** Thick lines represent actual rates; thin lines represent projected rates. Crude rates in full line; age- standardised rates in dashed line. Male (M) in blue; female (F) in red.

Table 2 presents the actual age-standardized rates and number of cases for 2005-2009 and those projected for 2015-2019 in Switzerland by cancer site and by sex. Figure 1 presents actual and projected rates for all cancer and individual cancer sites. Both crude and age-standardized rates are presented. The change in age-standardized rates approximates to the change in risk, while the change in crude rates includes, in addition, the effects of an ageing population.

**Table 2:** Actual age-standardized rates and number of cases for 2005-2009 and projections for 2015-2019 in Switzerland by cancer site and by sex

**Cancer site**	**Male**							**Female**						
	**Age standardised rates (per 100'000)**		**Number of cases (per year)**		**Change**			**Age standardised rates (per 100'000)**		**Number of cases (per year)**		**Change**		
	**2005-2009**	**2015-2019**	**2005-2009**	**2015-2019**	**Overall**	**Due to change in risk**	**Due to change in population**	**2005-2009**	**2015-2019**	**2005-2009**	**2015-2019**	**Overall**	**Due to change in risk**	**Due to change in population**
Oral Cavity & Pharynx	17.15	14.53	738	820	11%	-17%	28%	6.37	7.04	312	430	38%	18%	20%
Stomach	10.41	7.89	474	478	1%	-35%	36%	5.00	4.55	306	330	8%	-17%	25%
Colon, Rectum	49.34	45.76	2271	2908	28%	-9%	37%	30.55	28.47	1809	2084	15%	-9%	24%
Lung, Bronchus, Trachea	54.43	45.87	2460	2791	13%	-22%	35%	26.70	30.83	1380	2036	48%	26%	22%
Skin Melanoma	23.81	28.54	1053	1621	54%	25%	29%	21.06	24.01	1007	1341	33%	17%	16%
Breast								109.88	99.33	5388	5870	9%	-10%	19%
Corpus Uteri, Uterus NOS								16.84	16.80	880	1040	18%	-4%	22%
Ovary								11.28	9.68	584	622	6%	-15%	22%
Prostate	130.34	130.34	5900	8083	37%	0%	37%							
Bladder	18.73	15.38	885	1033	17%	-24%	41%	4.53	4.26	289	344	19%	-8%	27%
Thyroid	3.55	4.29	149	216	45%	27%	18%	9.26	11.81	406	586	45%	32%	12%
Non Hodgkin Lymphoma	17.55	19.11	779	1116	43%	12%	31%	12.61	14.07	688	935	36%	14%	21%
Other cancers	120.08	118.14	5295	6845	29%	-1%	31%	70.49	69.18	3864	4658	21%	-1%	22%
All Cancers except keratinocytic skin cancers	445.40	429.85	20005	25910	30%	-5%	34%	324.59	320.56	16913	20359	20%	-1%	21%

The predicted cancer burden in 2015-2019, except for non-melanoma skin cancers, is of 46269 new cases/year. Most importantly, the total number of cancer cases for men goes from 20005/year in 2005-2009 to 25910/year in 2015-2019, and for women from 16913 to 20359. The increase among men amounts to +30% with +34% due to the impact of demographic changes and a reduction of -5% due to changes in cancer risk. Among women, the overall increase is of +20%, basically entirely due to the demographic change, while almost no change in cancer risk is predicted (-1%). For all the cancer sites, an increase in the number of cases from 2005-2009 to 2015-2019 is predicted in both sexes. This increase is always attributable to the demographic changes and only for some sites to an increase of cancer risk as well. For many sites we observe a decrease in the cancer risk, however, this decrease is usually not strong enough to outweigh the increase due simply to the demographic growth.

The standardized incidence rates for all cancers combined (excluding non-melanoma skin cancer), are predicted to slightly decrease among men (from 445 to 430) and remain unchanged among women (from 325 to 321). Age-standardized rates show different patterns according to individual sites.

Rates are expected to decrease during the next years for stomach, colorectal and bladder cancer for both sexes, oral cavity & pharynx and lung cancer for males, breast, and ovary cancer for females (Figure 1, dashed lines). Among men, the largest increase in the burden is observed for melanoma (+54%), thyroid (+45%), non-Hodgkin lymphoma (+43%), and prostate (+37%). Prostate cancer will contribute the largest number of cases (8083) followed by colorectal cancer (2908) and lung cancer (2791). For women, cases of lung and oral cavity and pharynx are projected to increase by +48% and +38%, respectively; those of thyroid by +45% and non-Hodgkin lymphoma by +36%. The sites with the most cancer predicted are breast, with 5870 cases, followed by colorectal and lung with over 2000 cases each, melanoma with 1341 cases and corpus uteri with 1040 cases.

## Discussion

In this article we present, for the first time, projections of the future burden of cancer for all of Switzerland up to 2019.

Although the age-adjusted incidence rates for all cancers combined are expected to decrease slightly between 2009 and 2019 for men and stabilize for women, the number of cases is predicted to increase substantially in both sexes. Most of the increase will be driven by the demographic changes occurring in the Swiss population, in terms of its growing and ageing. In 2015-2019 the overall annual cancer burden is predicted to be 46269 new cases in Switzerland, corresponding to an increase of 30% among men and of 20% among women compared with 2005-2009. In terms of absolute numbers, the largest increase is expected for cancers of the prostate, with 8083 cases, and breast, with 5870 cases per year, followed by colorectum and lung with almost 5000 cases each, and melanoma with 2962 cases.

Results from cancer burden projections produced in other industrialized countries show largely similar patterns to ours. Projections in the USA show an increase by 26% of all cancers among men and by 18% among women from 2010 to 2020 [12]. Similarly to ours, the incidence of all cancers combined in the UK started to level off after a few decades of rise in 1998-2007 and is projected to fall by 1% in males and by 1.9% in females from 2007 to 2030. At 2020 the demographic changes will be responsible for an increase in the number of cases by 19% in men and 13% in women [13].

While the overall pattern of increase in the burden of cancer is comparable, there are important differences in predictions for some specific cancer sites. For lung cancer among men, for example, we project in Switzerland a decrease in the age-standardized rates with a relatively small increase in the number of cases (+13%) as compared to the increase projected in the UK and US (+17% and +29%, respectively). Similarly, among women we predict an increase in age-standardized rates and a particularly strong increase in the new cases, +48%. However, the number of lung cancer cases among women in the UK and in the USA is expected to increase by only +16% and +23%, respectively [12,13]. A much larger increase in the number of cases of melanoma, non-Hodgkin disease, prostate and thyroid cancer is also expected in Switzerland, as compared to the UK and USA [12,13]. The reasons for these disparities are difficult to explain and may in some part be due to differences in data modelling. However, part of the difference could be attributable to differences in the intensity of diagnostic procedures between the countries as well as to differences in the exposure to risk factors, preventive measures and screening. For example, a reduction in smoking prevalence has been associated with the decrease in cancer of the lung and oral cavity and pharynx observed in several countries (England, Canada, Nordic countries) [7,10,14]. In Switzerland a smoking ban has been set in place for public places only since 2010, much later compared to other countries, and different smoking behaviours may explain some of the differences observed in lung cancer projections with the other countries.

The Swiss health system is particularly strong in the diagnosis and treatment of cancer patients, which translates into internationally recognized high survival rates. However, the system is rather weak in prevention and health promotion, with spending on health promotion and prevention at 2.3% of total health expenditure, lower than the average of 2.7% for all countries of the Organisation for Economic Co-operation and Development [3]. The highly decentralised nature of the Swiss health system has historically made it difficult to deliver broad-based policies to address major chronic disease risk factors, such as tobacco smoking, alcohol abuse and obesity. Similarly, implementing cancer prevention measures with a proven value has been slow; for example, national screening programmes for breast cancer or colon cancer are not in place yet. However, with the confederation recently strengthening its role in health promotion and policy programs, there are new opportunities for improving the impact of such measures.

Future predictions depend on multiple assumptions, but the basic premise is that past trends will be carried forward into the future. Any changes in these trends will mean that the projections will not be realized. As suggested by Møller et al, [7] we modified this assumption by attenuating the ‘drift’ component of the observed changes in rates by 25% and, in cases of a significant departure from a linear trend we used only the trend in most recent 10 years, instead of the average of the whole period, to project the drift component. The model used in this paper has been tested and validated in the Nordic countries, in Canada, the UK and other countries and a set of conditions found to produce the most accurate predictions has been applied [7,13,14].

The effects of screening activities are difficult to account for in cancer projections, and this is especially true for Switzerland since screening programmes are not uniformly applied to the whole country and their initiation is not necessarily simultaneous. To minimise the impact that the high levels of PSA screening observed in the nineties had on cancer trends for prostate cancer we chose to use the 2005-2009 incidence rate constant, as already done by other authors [10,11]. For breast cancer, to take into account possible changes due to the implementation of screening programmes we modelled the rates separately for women in screening age from the others.

Another potential problem is due to the fact that Swiss cancer registries only partially cover the national population, and that the coverage was not uniform throughout the period considered for model fitting. The trends used for our predictions were derived from NICER national figures extrapolated from actual data recorded in cantons covered by cancer registration. NICER calculates the average incidence rate in each linguistic region by pooling cases from the relevant registries and their populations. This rate is determined separately by five-year age group, sex and cancer site. It is applied to the entire linguistic region assuming homogeneity of data between the geographical areas that are covered and those that are not covered. The Swiss estimate published by the Federal Statistical Office and NICER corresponds to the sum of estimated cases for each language region. Therefore, the estimates take into account the specificity of the geographical areas. However, at the end of 2009 cancer registration coverage was 61% of the whole country, with good representation of all major language regions at almost 100% for French-speaking Switzerland, 100% for the Italian-speaking region and 40% for the German-speaking region [4]. The decreasing reliability of cancer predictions over time is also related to the decreasing reliability of the population predictions. For population projections we have chosen the reference scenario reproducing the demographic changes observed at the beginning of the 21st century. We did not use those scenarios that increase the aging population or combine assumptions most favourable to population growth such as an increase in migration and fertility. The scenario of increased aging would certainly amplify the number of cancer cases. Such a scenario must be kept into account in future assumptions. However, given the relatively short period of our projections, this choice should not have introduced any serious error in this study.

## Conclusions

The burden of cancer is expected to increase sharply in Switzerland over the next 10 years and, unless marked improvements in cancer therapy and/or prevention strategies emerge, the number of cancer deaths may also grow substantially. New and more effective preventive measures should be put in place to counteract the inevitable increase in the number of cancer cases. Targeting risk factors such as obesity, physical inactivity, and tobacco use may result in incidence rates that are lower than those predicted. These expected rates for all cancers combined and for individual sites will provide a benchmark against which to measure the impact of prevention strategies in Switzerland.

Measuring the disease burden helps also to estimate the future needs of health care resources. To address the expected impact of increasing cancer incidence on the Swiss health care system, several additional interventions should be planned to prevent a potential shortage of health professionals, to invest in the infrastructure needed to deliver cancer care and to avoid an inevitable increase in cancer costs.

Switzerland has a high level of economic development and Swiss residents benefit from universal coverage, high levels of access to services and proximity to health services. The challenge for the years ahead will be to develop a system that can respond to the changing health risks and care needs of Swiss residents.

## Abbreviations

NICER: National Institute for Cancer Epidemiology and Registration.

## Competing interests

The authors declare that they have no competing interests.

## Authors’ contribution

ER participated in the conception, design and coordination of the study, in the analysis and interpretation of data and drafted the manuscript. SG performed the statistical modelling, and helped to draft the manuscript. BP and RM participated in the conception and in the design of the study, and critically revised the manuscript. MU participated in the conception and in the design of the study, in the analysis and interpretation of data and helped to draft the manuscript. All authors read and approved the final manuscript.

## Pre-publication history

The pre-publication history for this paper can be accessed here:

http://www.biomedcentral.com/1471-2458/14/102/prepub
